# Clinical Decision Support Systems for Pressure Ulcer Management: Systematic Review

**DOI:** 10.2196/21621

**Published:** 2020-10-16

**Authors:** Sabrina Magalhaes Araujo, Paulino Sousa, Inês Dutra

**Affiliations:** 1 Medical Informatics Faculty of Medicine and Faculty of Sciences University of Porto Porto Portugal; 2 Nursing School of Porto Porto Portugal; 3 Health Information Systems & Electronic Health Records Center for Health Technology and Services Research University of Porto Porto Portugal; 4 Department of Computer Science Faculty of Sciences University of Porto Porto Portugal; 5 Artificial Intelligence for Health Care Center for Health Technology and Services Research University of Porto Porto Portugal

**Keywords:** pressure ulcer, decision support systems, clinical, systematic review

## Abstract

**Background:**

The clinical decision-making process in pressure ulcer management is complex, and its quality depends on both the nurse's experience and the availability of scientific knowledge. This process should follow evidence-based practices incorporating health information technologies to assist health care professionals, such as the use of clinical decision support systems. These systems, in addition to increasing the quality of care provided, can reduce errors and costs in health care. However, the widespread use of clinical decision support systems still has limited evidence, indicating the need to identify and evaluate its effects on nursing clinical practice.

**Objective:**

The goal of the review was to identify the effects of nurses using clinical decision support systems on clinical decision making for pressure ulcer management.

**Methods:**

The systematic review was conducted in accordance with PRISMA (Preferred Reporting Items for Systematic Reviews and Meta-Analyses) recommendations. The search was conducted in April 2019 on 5 electronic databases: MEDLINE, SCOPUS, Web of Science, Cochrane, and CINAHL, without publication date or study design restrictions. Articles that addressed the use of computerized clinical decision support systems in pressure ulcer care applied in clinical practice were included. The reference lists of eligible articles were searched manually. The Mixed Methods Appraisal Tool was used to assess the methodological quality of the studies.

**Results:**

The search strategy resulted in 998 articles, 16 of which were included. The year of publication ranged from 1995 to 2017, with 45% of studies conducted in the United States. Most addressed the use of clinical decision support systems by nurses in pressure ulcers prevention in inpatient units. All studies described knowledge-based systems that assessed the effects on clinical decision making, clinical effects secondary to clinical decision support system use, or factors that influenced the use or intention to use clinical decision support systems by health professionals and the success of their implementation in nursing practice.

**Conclusions:**

The evidence in the available literature about the effects of clinical decision support systems (used by nurses) on decision making for pressure ulcer prevention and treatment is still insufficient. No significant effects were found on nurses' knowledge following the integration of clinical decision support systems into the workflow, with assessments made for a brief period of up to 6 months. Clinical effects, such as outcomes in the incidence and prevalence of pressure ulcers, remain limited in the studies, and most found clinically but nonstatistically significant results in decreasing pressure ulcers. It is necessary to carry out studies that prioritize better adoption and interaction of nurses with clinical decision support systems, as well as studies with a representative sample of health care professionals, randomized study designs, and application of assessment instruments appropriate to the professional and institutional profile. In addition, long-term follow-up is necessary to assess the effects of clinical decision support systems that can demonstrate a more real, measurable, and significant effect on clinical decision making.

**Trial Registration:**

PROSPERO International Prospective Register of Systematic Reviews CRD42019127663; https://www.crd.york.ac.uk/prospero/display_record.php?RecordID=127663

## Introduction

### Background

A pressure ulcer is an injury resulting from tissue compression and inadequate perfusion to the skin and underlying structures, usually over a bony prominence [1,2]. Pressure ulcer management performed by health care professionals involves phases of prevention, classification, diagnosis, and treatment. The implementation in clinical practice of appropriate strategies for pressure ulcer prevention is indispensable for improving the quality of nursing care.

The clinical decision-making process in pressure ulcer care phases is complex, and its quality depends on both the professional's experience and the availability of accurate knowledge [3]. Decision making should follow evidence-based practices, represented by the management of individualized care for each patient and integrating the use of the best evidence from scientific research [4,5]. The decisions made by nurses should be based on their clinical judgment, with consideration of recommendations in pressure ulcer management guidelines and a view to appropriate clinical practice [1].

Evidence-based guidelines for pressure ulcer prevention and treatment are widely available but are often overlooked or complex to implement in clinical practices. Schaarup et al [6] point out that many randomized controlled trials have concluded that health care professionals are often forced to rely only on their experiences when making wound care decisions because of the low evidence base in studies.

In order to guide professionals in decision making and following recommended guidelines, health information technology that has been incorporated into the clinical workflow, such as clinical decision support systems, may be used. These electronic systems are designed to generate patient-specific assessments or recommendations by comparing characteristics with a knowledge base to directly assist health care professionals in clinical decision making [7]. These systems can be classified into 2 types: (1) knowledge-based clinical decision support systems, expert systems based on inference mechanisms, and (2) nonknowledge-based clinical decision support systems, an inductive system with the application of artificial intelligence (machine learning), such as the use of artificial neural networks [8]. The main methodologies for clinical decision support systems are machine learning, knowledge representation, visualization techniques, and text mining [9].

Knowledge acquisition for these systems is related to the identification and assessment of the best available knowledge [3], making their effectiveness dependent on high-quality clinical research evidence that is up-to-date, easily accessible, and interpretable by computers [4]. The use of clinical decision support systems, in addition to assisting decision makers, can increase the quality of care provided [6,8,10] and reduce errors [8,10,11]. However, there is still limited evidence available on the widespread use of these systems [12], and the quality or relevance of research evidence may restrict their effectiveness [4].

### Objective

The purpose of this systematic review was to identify the effects of nurses using clinical decision support systems on clinical decision making for pressure ulcer management. Evaluation of these effects can clarify whether the incorporation of these systems in the workflow improves clinical nursing practice and nurses' knowledge.

## Methods

### Protocol Registration

This systematic review was conducted in accordance with recommendations by the Preferred Reporting Items for Systematic Reviews and Meta-Analyses (PRISMA) [13]. A protocol was developed to guide this review and was registered in the International Prospective Register of Systematic Reviews (PROSPERO CRD42019127663) [14].

### Search Strategy

The literature search was conducted in April 2019 on 5 electronic databases: MEDLINE/PubMed, Scopus, Cochrane, Web of Science, and CINAHL. The search strategy is reported in detail in Multimedia Appendix 1. Search results were exported and managed in EndNote (Clarivate Analytics). Reference lists of eligible articles were also screened manually for additional studies.

### Study Selection

In the first selection phase, studies were screened by assessing titles, abstracts, and keywords, after removing duplicates. The second phase of the full-text review was independently performed by 2 reviewers applying predefined inclusion and exclusion criteria. Eligibility criteria are presented in Textbox 1. The study design of the articles was not limited to high-quality randomized trials to increase the sample of clinical decision support systems publications on pressure ulcers. Qualitative, quantitative, and mixed method studies were included. There was no restriction on the year of publication.

Articles were reviewed by 2 nurses (SA, PS), and using the criteria, those evaluated as appropriate were included. Any disagreement between the reviewers was resolved by consensus or by a third author (ID) through discussion. Cohen κ statistic was calculated to quantify the agreement between reviewers.

**Eligibility criteria.**

**Inclusion criteria**

- described a computer-based clinical decision support systems used by health care professionals for pressure ulcer management
- addressed a clinical decision support systems that generated patient-specific recommendations

**Exclusion criteria**

- studies that were not written in English
- systems developed to aid teaching only and not to clinical practice
- clinical decision support systems for use on skin lesions or wounds other than pressure ulcers
- clinical decision support systems for use on a smartphone or any other device than the computer
- clinical decision support systems that only generated evaluation results, without specific recommendations
- clinical decision support systems that have not been evaluated or implemented in a real clinical setting

### Data Extraction

First author, journal of publication, year, country, study design, aim, pressure ulcer phase (prevention, classification, diagnosis, treatment) for the clinical decision support system application, health care setting involved, participants, type of clinical decision support system and guidelines used, main function of the clinical decision support system, identified evidence, and results of included studies were extracted by one reviewer and confirmed by another.

### Clinical Decision Support Systems Classification

Two types were considered in the classification of the clinical decision support systems [8]: knowledge-based (deductive system based on inference engines, usually in the form of *if-then* rules) and non–knowledge based (inductive system with application of artificial intelligence). Another classification used in this review divided the clinical decision support systems into 5 groups, according to their methodologies: machine learning (artificial neural networks, logistic regression, support vector machines), knowledge representation (ontology-based systems, guideline-based, fuzzy logic), information visualization (visualization algorithms to encode abstract concepts and information), text mining (natural language processing and information retrieval), and multipurpose (various attributes and characteristics of existing domains, includes decision trees and Bayesian logic) [8,9].

### Study Quality

The methodological quality of included studies was assessed using the revised version of the Mixed Methods Appraisal Tool (MMAT) [15]. The MMAT contains a checklist with 5 questions to assess methodological quality for each study design category, defined by MMAT with a number from 1 to 5: (1, qualitative; 2, quantitative randomized controlled trials; 3, quantitative nonrandomized; 4, quantitative descriptive; 5, mixed methods). Each criterion must be answered as “yes,” “no,” or “can't tell.” The studies were analyzed separately and were considered to be of high quality when meeting 100% (5/5) of the criteria, considerable quality with 80% (4/5) of the criteria, moderate quality with 60% (3/5) of the criteria, low quality with 40% (2/5) of the criteria, and very low quality with 20% (1/5) of the criteria.

## Results

### Search Results

**Figure 1:**
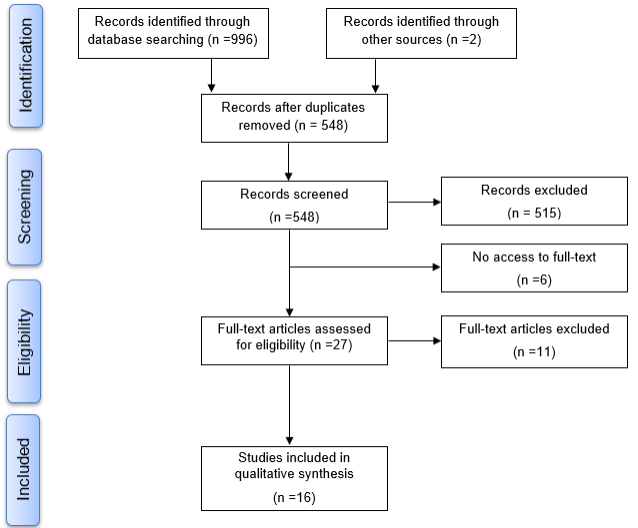
Flow diagram of the selection process.

The search strategy yielded a total of 996 articles, and 2 additional articles were identified manually, resulting in 998 articles. After removing duplicates, 548 articles were analyzed in the first phase, in which, 515 articles were excluded; therefore, 33 articles were eligible for the second phase. Access to 6 articles was not possible, and 11 were excluded for different reasons (see Multimedia Appendix 2). Hence, 16 studies [16-31] met all the eligibility criteria and were included in this review. A flow diagram of the selection process is presented in Figure 1.

### Kappa Statistics

When analyzing the selection of the 16 studies included in the qualitative synthesis, the value obtained from the Cohen κ coefficient was 0.67. This value represents substantial strength of agreement between reviewers [32].

### Study Quality

In assessing methodological quality using MMAT [15], included studies were classified according to category, and each group was analyzed separately for quality assessment. Methodological quality results are presented in Multimedia Appendix 3. Of the 16 studies, 3 used qualitative research, only 1 was a randomized controlled trial, 8 studies used a nonrandomized quantitative approach, and 2 studies used mixed methods. A total of 5 studies were rated as high-quality, 6 studies were rated as considerable quality, 2 studies were rated as moderate quality, and 1 study was rated as low quality. There were no studies rated as very low quality; however, 2 articles [16,18] did not receive a classification because all 5 criteria analyzed obtained a “can't tell” answer. These 2 studies did not meet any quality criteria in their study category. Both described clinical decision support systems for the care of pressure ulcers but did not describe methodology used for analysis and data collection, which made assessment with MMAT unfeasible. No study was excluded based on quality assessment.

### Study Characteristics

General characteristics of included studies are shown in Multimedia Appendix 3. Included studies were conducted between 1995 to 2017 in the following countries: United States of America [16,17,20,21,25-27], Italy [18], Canada [19], Norway [22-24], South Korea [28], Belgium [29], and Singapore [30,31]. The studies were published in 9 different journals and in symposium proceedings, most of which related to health informatics (9/16, 60%), followed by nursing sciences (3/16, 20%). The clinical decision support systems were implemented to support nurses' clinical decisions in multiple clinical and health care settings such as nursing homes [22-24,26,27,29]; hospital inpatient units (medical-surgical) [16-18,20,21,30,31]; acute, home, and extended care [19]; intensive care [28]; and long-term care facilities [25]. The clinical decision support systems were used in pressure ulcer prevention [18,20,21,25-29], prevention and treatment [16,17,19], pressure ulcer prevention and evaluation of nutritional status [22-24], and treatment [30,31]. Interventions in the studies were based on the implementation of clinical decision support systems in clinical practice with follow-up periods ranging from 1 month [18] to 12 months [19,27] or more [31].

All included studies describe knowledge-based systems—13 out of 16 systems were classified as knowledge representation, with methodologies such as decision rules (*if-then* model) [20,30,31], guideline modeling language (GLIF, Guideline Interchange Format) to validate the logic of enhanced decision rules [21], or clinical practice guidelines represented through the graphic editor GUIDE, written in Java [18]; and 3 out of 16 systems were classified as multipurpose, with 2 using decision trees [30,31] and 1 using a Bayesian network model [28].

In 7 out of 13 systems classified as knowledge representation, the clinical decision support systems were developed based on Agency for Healthcare Research and Quality guidelines for pressure ulcer prevention and treatment [16-19,25-27]. The Braden Scale [16,19-21] and the Risk Assessment Pressure Scale [22-24], both for pressure ulcer risk screening, also appear as evidence bases. The Pressure Sore Status Tool [19], an instrument for pressure ulcer evaluation; the American Medical Directors Association guidelines for pressure ulcer prevention [25,26]; and opinions of pressure ulcer experts on the decision-making rules of the clinical decision support systems [16,18-21,29] were other knowledge described in the articles. In addition, literature reviews to identify the best evidence for pressure ulcer care were also used to create the systems [16,19,23,28,29,31]. The classification, evidence base, and function of the clinical decision support systems are detailed in Multimedia Appendix 4.

### Clinical Decision Support Systems in Analysis

#### Effects on Nurses' Clinical Decision Making

Few studies evaluated the effects on nurses' decision making. Nurses acknowledged advantages after a month of testing the implementation of a computerized guideline for pressure ulcer prevention in a general medicine ward; users reported that the daily prevention work-plans generated by the clinical decision support systems and the detailed storage of actions were useful in making decisions for planning patient discharge [18].

On the other hand, nurses at a public tertiary hospital in Singapore reported low credibility and confidence in the implemented clinical decision support systems [30]. This assessment, influenced by the workplace culture, had consequences for the adoption of the system and for nurses' decision making. Instead of what was recommended by the clinical decision support system, many nurses preferred to follow their past experiences or opinions of leaders and wound experts when determining the treatment modalities for the wound [30]. The same was observed in the study by Clarke et al [19] in which some nurses perceived the care plans generated by the clinical decision support systems as elementary, preferring to trust on their own assessment skills.

Regarding the knowledge acquired by professionals after the implementation of clinical decision support systems, which could have a positive effect on decision-making skills in the care of pressure ulcers, the results were paradoxical. Clarke et al [19] observed an increase in knowledge about pressure ulcers prevention, treatment strategies, resources required, and the importance of interdisciplinary teams in the daily planning of interventions. However, in the studies by Zielstorff et al [17] and Beeckman et al [29], the results showed no significant improvement in nurses' knowledge about pressure ulcer prevention and treatment, when comparing the knowledge assessment instrument results applied to health care professionals in the intervention and control groups, before and after the implementation of clinical decision support systems.

#### Factors That Influence the Use or Intention to Use and Successful Implementation in Clinical Practice

Nurses had favorable attitudes toward use when a clinical decision support system [28] was implemented in an intensive care unit using data from the electronic health record to predict hospital-acquired pressure ulcers. In nursing homes, some nursing personnel who were comfortable with computer technology evaluated the use of clinical decision support systems with positive feedback, while others expressed resistance to use [23]. In the studies, various reasons that influenced nurses' adoption of the systems to support clinical decision making in pressure ulcer care were observed. Professional, organizational, and software-design barriers affected the use of clinical decision support systems by nurses. The main advantages and difficulties of using the clinical decision support systems that were assessed by users are presented in Textbox 2.

**Advantages and difficulties assessed by users in using clinical decision support systems to care for pressure ulcers.**

**Advantages**

- Easy to use [17,18,23,30]
- Detailed documentation [18,19,24,25]
- Improved planning [18,19,25]
- Workload assessment [18]
- Useful at the patient discharge [18]
- Education [18]
- Facilitates handing on duties to the next shift nurses [18]
- Implementing and following the protocols [16,29]
- Improved the recording of nursing assessments and comprehensiveness [24,25]

**Difficulties**

- Lack of flexibility [18,23]
- Lack of logical flow [23,30]
- Lack of time for data input [17,18,23]
- Lack of computer skills [19,23]
- Lack of training [19,23]
- Lack of computer infrastructure [19]
- Lack of information about the clinical decision support systems implementation [23]
- Resistance to use computers [23]
- Workplace culture [30]
- Lack of trust and credibility in clinical decision support systems [30]
- Frustration with clinical decision support systems use [19,30]

The factors associated with successful clinical decision support system implementation in clinical practice were involvement of the administrator or head of nursing in the process [25,26], emphasizing the importance of leadership that was actively engaged; the presence of an internal champion [26] as a key nurse [29], who can be a persuasive leader as the force for change; and participation of an interdisciplinary team, facilitators, and a quality improvement team [25,26,29] in the health care organization. In addition, consideration of clinical workflow [18,31], training and previous education activities for professionals on the use of clinical decision support systems [19,22-25,28,29] and the importance of preventing pressure ulcers [28,29] performed before implantation of the clinical decision support systems were also described in the articles as factors associated with success.

### Clinical Effects on Pressure Ulcer Incidence and Prevalence

Preliminary results in one study [16], indicated a significant reduction, from 7% to 2%, in pressure ulcer incidence in the case units, 6 months postimplementation of a clinical decision support system for pressure ulcer prevention in an American hospital. In the study by Olsho et al [27], this clinical effect occurred in nursing homes that jointly implemented 4 components (nutrition, weight summary, priority, trigger summary), avoiding approximately 2.6 pressure ulcers per 100 patients per month (*P*=.035).

In 7 long-term institutions that implemented a clinical decision support system [25], there was a decrease in the percentage of high-risk residents with pressure ulcers from 13.0% (before implementation) to 8.7% (12 months after implementation), with a combined reduction of 33%. However, quality control decreased in 5 facilities and increased slightly in 2 facilities that did not implement all the system reports.

In the intervention group of an intensive care unit, adoption to the clinical decision support systems [28] for pressure ulcer prevention allowed a 21% to 4% reduction in the prevalence of hospital-acquired pressure ulcer and decreased the length of stay by approximately one-third (7.6 to 5.2 days). Beeckman et al [29] also observed a decrease in the prevalence of pressure ulcers after using a clinical decision support systems in the experimental group. The result was clinically meaningful but nonstatistically significant. Therefore, no overall significant effect was found on pressure ulcer prevalence [29].

## Discussion

### Principal Results

As for the impact on nurses' knowledge with the use of clinical decision support systems, only 3 included studies evaluated this effect and obtained paradoxical results. There was no description of the time of data collection to assess knowledge, nor of the type of assessment used, in the study [19] that identified an increase in nurses' knowledge after the intervention. In studies in which this effect was not identified, few nurses participated in the posttest [17], and there were limitations in the knowledge questionnaire applied before and after the clinical decision support systems implementation [29]. The assessment instrument for nurses was used with health care professionals who had no nursing education background and may have been too difficult, resulting in low scores on the instrument [29].

Evidence of the effect of clinical decision support systems on clinical knowledge is still insufficient, with evaluations carried out after short periods of system implementation that may not demonstrate measurable effects [17] as well as with small sizes in the assessed sample.

As for the factors that influenced the use or intention to use clinical decision support systems and the success of implementations in included articles, the professionals played important roles in the process. Several professional and organizational barriers were identified in the adoption of the clinical decision support systems, as well as in nurses’ relationships with the use of the systems. Relying on their own assessments, instead of the recommendations generated by the clinical decision support systems, was an observation found only in studies that analyzed the use of systems in pressure ulcer treatment.

Gerrish et al [33] reported that nurses rely heavily on communication with colleagues and their personal experience rather than formal sources of knowledge. Dowding et al [34], also described that nurses report relying on their experience when dealing with tasks in which decisions seemed more familiar and using the clinical decision support for situations with which they had little experience.

The interaction between the nurse and the technology must be considered by involving end users during all stages of the implementation and in evaluations of the system [34,35]. The user's computer knowledge and training on the clinical decision support systems also directly affected the adoption of the systems. Ammenwerth et al [36] identified that a professional's computer knowledge and previous acceptance of the nursing process were 2 factors that were significant predictors of user acceptance of computerized nursing systems. The other factors observed were the fit between the nursing workflow and the functionality of the system [36].

An important basis for clinical decision support system design is an understanding of the clinical care process and local workflow. Decision support can be provided continuously throughout the care process, at the most effective level of nursing care (from the user's initial assessment to the outcome evaluation) [37]. The use of clinical decision support systems allowed increased compliance with pressure ulcer prevention protocols, improving professional attitudes, in addition to encouraging more complete documentation and more comprehensive nursing assessments [24,25]. The other benefits included consistency in the quality of nursing care and greater access to information on best practices [38].

Clinical decision support system implementation must be based on models of technology adoption, evidence-based practices, and conceptual models in nursing practice. The success of clinical decision support system implementation will clearly depend on the analysis of critical success factors, and modeling efforts should allow for the broadest and most effective use of the systems [39]. Only 4 studies [19,23,28,29] addressed the use of some model or conceptual framework as a guide, organizing implementation strategies and elucidating the variables found.

Clarke et al [19] used 5 phases of the adoption of innovation [40] and 5 factors influencing the rate of adoption of innovations [41] models; Fossum et al [23] applied the Task Technology Fit model [42]; to measure the user's attitude toward the system, Cho et al [28] used the United Theory of Acceptance and Use of Technology [43] model questionnaire; and Beeckman et al [29] used a model for effective implementation [44].

To trigger improvement in nursing practice, it is important that clinical decision support systems have following characteristics: automatic provision of decision support, facilitating clinical practice and decreasing the professional's effort; provision of recommendations, rather than just evaluations; and provision of decision support at the time and location of clinical decision making [45,46]. According to Kawamoto et al [45], nursing practices improved significantly in 94% of the analyzed trials when all these characteristics were present in the clinical decision support system.

Automatic prompting in clinical decision support systems can improve integration into the workflow and provide the opportunity to correct inadvertent deficiencies in care [47]. The decision support system [16] that used an alert logic had a positive impact in reminding nurses about the completion of each patient's processes. Only 6 out of 50 admissions were completed on the system without prompting alerts. The availability of this tool in clinical decision support systems affects the performance of professionals [10,47]. However, these reminders should be relevant to the patient’s profile so that the user does not reject them [10]; interfaces with many alerts can generate frustration when using the clinical decision support systems, decreasing workflow, quality, efficiency, and safety in providing patient care [10].

As for the clinical effects from using a clinical decision support systems, the reduction in the pressure ulcer incidence was considered to be of low evidence. One of the studies [16] with this finding did not meet any MMAT quality criteria in its study category. In the other [27], the analysis was subject to several important limitations, and there was an imprecision associated with the estimate when the 95% confidence interval was applied.

In reducing pressure ulcer prevalence, there was a possible bias in the study by Cho et al [28] from the long time elapsed between the intervention and the observation, which may have positively influenced the results of both the reduction of pressure ulcers and the length of intensive care unit stay [48]. The study by Fossum et al [22] showed no effect on patient outcomes in relation to pressure ulcer risk and prevalence. However, all the groups that were evaluated had smaller samples than those recommended by power analysis calculations. The positive clinical effects shown in the included studies were mostly clinically significant but without statistical significance.

Assessing and interpreting the clinical effects generated by the clinical decision support system intervention, as well as obtaining results with strong evidence in clinical practice, can be a difficult task. This can happen because clinical decision support systems are knowledge-based, using, for example, expert opinions and prevention scales when creating the algorithms. There is still no strong evidence that the risk of developing pressure ulcer decreases with the use of pressure ulcer risk assessment instruments (such as the Braden scale) when compared to less standardized risk assessment based on nurses’ clinical judgment [49].

Thus, if the evidence from the system's knowledge base has scientific limitations, the clinical effects generated by clinical decision support system may also be limited. There is also a difficulty in identifying, in the widely available literature, the best knowledge to be used to create this type of system [3]. In this way, clinical decision support systems will only be able to facilitate the implementation of evidence-based care when the systems can follow the literature in identifying high-quality studies and incorporate the best evidence to generate more appropriate recommendations [4].

### Limitations

This systematic review was limited by the eligibility of heterogeneous studies, publication bias, location bias, and nonconducted meta-analysis. There was a plurality of methodological approaches, not limited to randomized controlled trials. However, this is often a necessary approach to expand the understanding of clinical acceptance influenced by clinical decision support system development and deployment [50].

In addition, most of the studies evaluated were not randomized, with an inherent risk of bias. However, the quasi-experimental design is often used in many medical informatics articles to evaluate the benefits of specific interventions when it is not logistically feasible or ethical to conduct a randomized controlled trial [51]. Finally, the analysis of the results was limited, with some included studies that published only preliminary results [16-19].

### Directions for Future Studies

Effects of clinical decision support systems used by nurses in the management of pressure ulcers lack results of strong evidence in the literature. It is necessary to carry out studies that prioritize better adoption and interaction of nurses with these systems by making this the focus during the development of clinical decision support systems and in planning implementation strategies, as well as having studies with representative samples of health care professionals, randomized designs, and the application of assessment instruments appropriate to the professional profile and consistent with the health care organization. Longer periods should be used for the evaluation of the effects of the clinical decision support systems, which may have a more real, measurable, and significant effect on clinical decision making. In addition, these studies should be accompanied by the creation and implementation of systems based on recommendations and successful models, for better adoption by nurses to clinical decision support systems in the pressure ulcers treatment.

### Conclusions

Evidence in the available literature is still insufficient regarding the effects of nurses who use clinical decision support systems on clinical decision making for pressure ulcer prevention or treatment. No significant effects were found on nurses' knowledge following the integration of clinical decision support systems into workflows, with assessments made for a brief period of up to 6 months of implementation. Clinical effects, such as outcomes in the incidence and prevalence of pressure ulcers, remain limited, and most were clinically significant but nonstatistically significant.

## Appendix

Multimedia Appendix 1 The search strategy.

Multimedia Appendix 2 Reasons for records exclusion in the screening and eligibility phase.

Multimedia Appendix 3 General characteristics of the included studies.

Multimedia Appendix 4 Characteristics of the clinical decision support systems described in the included studies.

## Abbreviations

MMAT: Mixed Methods Appraisal Tool
