# Supplementary material for: Clinical Decision Support Systems for Pressure Ulcer Management: Systematic Review
Source: JMIR Med Inform. 2020 Oct 16;8(10):e21621. doi: 10.2196/21621 (PMC7600011; doi:10.2196/21621)
Supplement: Multimedia Appendix 1 [file medinform_v8i10e21621_app1.pdf]

Multimedia Appendix 1. The search strategy.

Strategy	Population/Condition: Pressure ulcer
**#1**	*"Pressure ulcer" OR "pressure ulcers" OR "pressure wound" OR "pressure injuries" OR "pressure injury"*
	AND
	**Intervention: Clinical Decision Support System**
**#2**	*"Decision support systems, clinical" OR "Clinical Decision Support System *" OR "Clinical decision support" OR "clinical decision Making" OR "CDS" OR "CDSS" OR "algorithm" OR " Decision Making, Computer-Assisted"*
