# Supplementary material for: Clinical Decision Support Systems for Pressure Ulcer Management: Systematic Review
Source: JMIR Med Inform. 2020 Oct 16;8(10):e21621. doi: 10.2196/21621 (PMC7600011; doi:10.2196/21621)
Supplement: Multimedia Appendix 2 [file medinform_v8i10e21621_app2.pdf]

Multimedia Appendix 2. Reasons for records exclusion in the screening and eligibility phase.

Screening

- Context other than only PU[a]: 195
- Context other than CDSS[b]: 176
- Sensor or system of pressure detection/inclination on bed, cushion, or wheelchair: 49
- Image processing/mining, multispectral images: 29
- Guidelines: 18
- Algorithm not applied in CDSS computerized: 11
- Mobile application: 8
- Not in English: 8
- Systematic review: 5
- Care Dependency Scale: 4
- Study with animals: 4
- Letters/Editorial: 4
- Education tool: 2
- CDSS not applied in clinical practice: 1
- Protocol: 1

Records excluded: 515

Eligibility

- Secondary studies or abstracts of primary studies included: 7
- CDSS not applied in clinical practice: 3
- Non CDSS description: 1

Records excluded: 11

[a]Pressure ulcer
[b]Clinical Decision Support Systems
