# Supplementary material for: Clinical Decision Support Systems for Pressure Ulcer Management: Systematic Review
Source: JMIR Med Inform. 2020 Oct 16;8(10):e21621. doi: 10.2196/21621 (PMC7600011; doi:10.2196/21621)
Supplement: Multimedia Appendix 3 [file medinform_v8i10e21621_app3.pdf]

Multimedia Appendix 3. General characteristics of the included studies.

Study	Setting Participants	Intervention	Results	Discussion	MMAT[a]
Willson et al [16]<br><br>USA, 1995	Medical/ surgical units<br><br>3 cases units and 1 control unit	Implementation of the computerized protocol integrated with the information system previously used in the hospital.	Compliance to the prevention protocol increased with the computerized Braden Scale and the computer generated "reminder" alerts. Compliance to the treatment protocol has improved. The results showed a statistically significant decrease in the PU[b] incidence from 7% to 2%. The primary benefit of the information system has been to support the nursing staff in implementing and following the protocols.	Some nurses have not yet complied with the prevention protocol and some who continually ignored reminder alerts. The protocol team should have clarified expectations and implemented a process to evaluate nurses' acceptance and hold nurses accountable for compliance to the protocol. Reduction in PU rates with consequent cost savings due to reduced treatment and equipment costs in the hospital.	Non-randomized study<br>-
Zielstorff et al [17]<br><br>USA, 1997	Case: 20-bed inpatient orthopedic/ neurosurgery unit; 15 nurses; 12 nurses in the pos-testing<br><br>Control: 28-bed acute orthopedic unit specializing in trauma; 17 nurses; 9 nurses in the pos-testing	Experimental implementation of a decision support system for 21 weeks.	A 21-week exposure to the system did not affect nurses' knowledge and nurses' clinical decision making related to PU prevention and treatment. The system received an overall positive rating by end-users, both on the written structured survey and in face-to-face interviews.	There is no evidence that the system influenced results in the level of knowledge and clinical decision making of nurses in the experimental unit. Only preliminary findings in the study. The small number of participants, and the attrition of 47% of the control group, make definitive conclusions impossible.	Non-randomized study<br>3 (80%)
Quaglini et al [18]<br><br>Italy,<br><br>2000	General medicine ward<br><br>Data from 40 patients	Implementation of a computerized guideline integrated into the EHR[c] and within the clinical routine for 1 month.	As regards the educational tool, it had been judged as rich and easy to use. Nurses complained about a certain rigidity of the computerized guideline in assessing the patient's risk, and in continuously asking for task completion. They required minimizing the	Users appreciated the documentation generated by the computerized guideline, which facilitated handing on duties to the next shift nurses, useful at the patient discharge and was also a means for assessing the nurse workload required by	Qualitative study<br>-

			time spent in data input by generating some default values.	the prevention program.<br>No data are available yet to evaluate the benefits on patient outcomes because the system has been installed for a too-short time.	
Clarke et al [19]<br><br>Canada, 2005	7 health care organizations (acute, home and extended care)	Implementation of the computerized clinical practice guidelines project in the 2-year period between the preparation and evaluation phase.	Increased knowledge about PU prevention, treatment strategies, resources required, and the role of the interdisciplinary team.<br>Barriers: lack of leadership, the time required to acquire computer skills and difficulties in using the CDSS[d].	The effectiveness of the implementation depends nursing leadership, organizational structures, and informatics.<br>The CDSS permits changes in the plan by nurses. Prevalence and incidence estimates are available on an ongoing basis.<br>Only some search results are available.	Qualitative study<br>1 (40%)
Kim et al [20]<br><br>USA, 2010	Medical/ surgical units<br><br>Data from 39 patients	Develop an automated pressure ulcer risk prototype assessment tool and test its performance with a small set of patient data.	The CDSS and the nurses showed varying levels of agreement (from “slight” to “substantial”) on assigning scores for the six parameters of the Braden scale.	Further studies should be conducted to elucidate the origins of the differences in the agreement. The CDSS had a poor performance in the decisions about the parameter of moisture exposure and nutritional status.<br>Nurses could accept or modify the scores attributed by the CDSS.<br>The use of better quantifiable descriptions will probably increase the consistency, there were free texts with nominal and numerical values and coded lists for algorithm analysis.	Mixed methods study<br>5 (100%)
Choi et al [21]<br><br>USA, 2013	Data from 99 patients: Cardio General unit (n = 20), Musculoskel etal	Enhancement of decision rules and new validation of the CDSS.	After the changes in the system, the level of agreement between the CDSS and the expert PU assessments was "very good" (kappa= 0.83).<br>The sensitivity and	The successful enhancement of decision rules has increased the generalizability and performance of the CDSS. Need to	Non-randomized study<br>3 (80%)

	unit (n = 20), Stroke unit (n = 30), Brain Injury unit (n = 10) and Spinal Cord Injury unit (n = 19).		specificity were 86.8% and 90.3%.	improve the moisture parameter of the Braden scale. There was an increase in the level of concordance, but with limitations still of the tool.	
Fossum et al [22] Norway, 2011	15 Nursing homes 46 units; 491 patients before and 480 after the intervention	One control group and two intervention groups with educational sessions about the PU prevention, one group also with the CDSS implementation integrated into the EHR.	A significant decrease in the proportion of malnourished residents with the CDSS implementation and no significant effects in relation to the PUs.	The result showed no other effect on residents' results in relation to the prevalence of PU and prevalence of malnutrition after the education and implementation of the CDSS.	Non-randomized study 3 (80%)
Fossum et al [23] Norway, 2011	4 Nursing homes 25 nursing personnel (Registered Nurses n=19, a Special Needs Educator n=1, Nurse Aides n=5)	Qualitative descriptive study to describe the facilitators and barriers in adhering to the CDSS for the care of nursing home residents.	Ease of use, usefulness and a supportive work environment were key facilitators of CDSS use. The barriers identified were lack of training, resistance to using computers and limited integration of the CDSS with EHR.	Organizations must invest more resources in educating nursing personnel on the seriousness of PUs, providing specialized CDSS training, and ensuring time in the workday to use the system.	Qualitative study 1 (100%)
Fossum et al [24] Norway, 2013	15 Nursing homes 46 units; data from 150 records before and 141 records after the intervention	Two intervention groups with educational sessions, one also with implementation of the CDSS for eight months. A group control without interventions.	More complete and comprehensive documentation of PU and malnutrition related nursing assessments and nursing interventions.	These results show that the use of CDSS and the educational program improved the registration of nursing assessments and comprehensiveness in the documentation of PU and nutritional problems. Nurses could add, delete, and change interventions. The CDSS required entry to all data items in the risk assessment. Limitation could be the non-randomized group design, which means that the nursing homes, and not the individual nurses, were the intervention units.	Non-randomized study 3 (80%)

Horn et al [25]<br><br>USA, 2010	11 LTC[e] facilities	Project facilitators assisted frontline multidisciplinary team to develop streamlined standardized CNA[f] documentation and weekly reports to identify high-risk residents and to integrate clinical reports into day-to-day practice and clinical decision making.	Reduction in numbers CNA documentation and improvements in completeness rates. Success in the facilities was associated with the participation of the director of nursing, multidisciplinary quality improvement team and team of facilitators. Percentage of long-stay high-risk residents with pressure ulcers decreased from 13.0% (before implementation) to 8.7% (12 months after implementation) in 7 project facilities.	The CDSS redesigned CNA workflow and documentation to include standardized data elements related to PU risk factors, integrated into daily practice, and provided clinical reports for staff to use in multidisciplinary communication, clinical decision making, and resident care planning.<br><br>Study facilities were voluntary and most had previous experience in quality improvement and making process changes using a multidisciplinary team approach.	Non-randomized study 3 (60%)
Sharkey et al [26]<br><br>USA, 2013	14 Nursing homes	Implementing the CDSS quality improvement for PU prevention program and integrating health information technology tools into practice at the unit level.	After at least 9 months of implementation effort, 36% of the nursing homes achieved a high level of system implementation. Associated factors: involvement from the administrator or director of nursing, participation of nurse manager, presence of in-house dietitian, staff educator and quality improvement personnel, presence of an internal champion, and team's openness to redesign.	Several factors contribute to the successful integration of a quality improvement program into the nursing home workflow. Facilities with many different quality problems may not be ready to implement a system focused on improving quality.<br><br>CDSS had different levels, with processes to be implemented gradually, to improve the quality and involvement of the team.	Non-randomized study<br><br>3 (60%)
Olsho et al [27]<br><br>USA, 2014	Case: 12 Nursing homes; 3463 residents<br><br>Control: 13 Nursing homes; 2698 residents	Employed an interrupted timeseries design to identify impacts of 4 core CDSS program components on resident PU incidence in nursing homes implementing the intervention.	Statistically significant reduction in PU incidence associated with the joint implementation of 4 core CDSS components (incidence rate ratio = 0.409;	The CDSS implementation was associated with reductions in PU incidence, approximately 2.6 PUs avoided per 100 residents per month. Adoption of 3 of 4 core	Non-randomized study 3 (100%)

			P = 0.035). Impacts vary with implementation of specific component combinations	components yields impacts of similar magnitude.	
Cho et al [28]<br><br>South Korea, 2013	ICU[g]<br><br>64 nurses<br><br>Case: 866 patients<br>Control: 348 patients	Implementing of the CDSS linked to the EHR for 6 months in the intervention group.	In the intervention group, the overall HAPU[h] prevalence rate fell from 21% to 4.0% and the length of stay shortened from 7.6 to 5.2 days.	Incorporated into the EHR, but interface on separate screen.<br>The Bayesian network model exhibited better prediction and case coverage than other modeling methods such as decision tree and regression models.	Non-randomized study<br>3 (80%)
Beeckman et al [29]<br><br>Belgium, 2013	4 Nursing homes<br><br>Case: 6 wards; 239 patients and 65 healthcare professionals<br><br>Control: 5 wards; 225 patients and 53 healthcare professionals	Two-armed randomized, controlled trial with a 16-week implementation intervention. The control units received lectures on PU prevention and the experimental units implemented the CDSS, including interactive education, reminders, monitoring, feedback, and leadership.	No significant improvement was observed in the prevalence of PU and knowledge of professionals. Improved preventive care with wheelchairs.	No overall significant effect was found on PU prevalence (Category II–IV). However, clinically meaningful (but non-statistically significant) differences were found between the PU prevalence figures in the experimental and the control arm.<br>Significant improvement in the attitudes toward PU prevention of professionals.<br>Reasonable to assume that the key nurse was a "clinical champion", a persuasive leader, being the force for change.	Randomized controlled trial<br>2 (80%)
Khong et al [30]<br><br>Singapore, 2015	Multidisciplinary nursing units in a tertiary hospital<br><br>14 nurses	To explore the clinical decision-making that influenced nurses' decisions to adopt the CDSS fully deployed for 10 months.	Eight themes were identified of nurses' clinical decision-making in adopting the system: the use of the CDSS, beliefs in the CDSS, the influences of the workplace culture, the extent of benefits, control over nursing practices, the use of knowledge, gut feelings, and emotions.	If the eight themes identified could be addressed and managed during the design and deployment of the CDSS, it could improve the adoption of the CDSS in clinical areas.	Qualitative study<br>1 (100%)
Khong et al [31]<br><br>Singapore, 2017	Nursing units<br><br>40 nurses	Adoption of an engineering cycle in four phases (knowledge acquisition,	The overall agreement exceeded 90% between the wound experts and the generated treatment modalities for	The CDSS has the potential for inculcating evidence-based practice, is cost-effective, and has	Mixed methods study<br>5 (100%)

	knowledge representation, knowledge Application, and knowledge evaluation) to design the construct base for the CDSS.	the choice of wound products, instructions, and alerts.<br>The CDSS serves as a just-in-time wound treatment protocol with suggested clinical actions for nurses, based on the best evidence available.	wide adoption. The success in the design, construction, and implementation of this CDSS lies in having strong leadership with multiple stake-holders' support, and solid financial support.<br>The design of future CDSS constructs should incorporate skin temperature, the presence of infection, and the type of exudate in the decision rule sets.

[a]Mixed Method Appraisal Tool. The number/percentage refer to design/quality [15].
[b]Pressure ulcer
[c]Electronic Health Record
[d]Clinical Decision Support Systems
[e]Long-term-care
[f]Certified nurse aide
[g]Intensive care unit
[h]Hospital-acquired pressure ulcer
