# Supplementary material for: Clinical Decision Support Systems for Pressure Ulcer Management: Systematic Review
Source: JMIR Med Inform. 2020 Oct 16;8(10):e21621. doi: 10.2196/21621 (PMC7600011; doi:10.2196/21621)
Supplement: Multimedia Appendix 4 [file medinform_v8i10e21621_app4.pdf]

Multimedia Appendix 4. Characteristics of the CDSS described in the included studies.

Study	Type of CDSS[a]/ Evidence base	CDSS function
Willson et al [16] (1995)	• Knowledge-based Representation of knowledge<br>• Braden scale, AHRQ[b], literature review and expert consensus	It automatically displays the interventions suggested by the protocol specific to the Braden Scale scores and proposes a treatment for the PU[c] stage, based on data computed by nurses. Generate alerts to review the scale.
Zielstorff et al [17] (1997)	• Knowledge-based Representation of knowledge<br>• AHRQ	Supports the nurse's development of patient-specific, guideline-based treatment plans for patients who have PU or are at risk for developing them.
Quaglini et al [18] (2000)	• Knowledge-based Representation of knowledge<br>• AHRQ, AISLEC[d] and nurses/physicians from the hospital test	Prepares a daily prevention work plan for patients at risk of developing PU, based on the data entered by professionals in the electronic patient record. It also has an educational tool to simulate patients.
Clarke et al [19] (2005)	• Knowledge-based Representation of knowledge<br>• AHRQ, PSST[e], Braden authors, experts, and literature review.	Nurses completed a Braden Scale skin assessment for all patients and a PSST for those who were found to have pressure ulcers. This information was entered into the computerized program and a plan of care generated.
Kim et al [20] (2010)	• Knowledge-based Representation of knowledge<br>• Expert nursing panel and Braden scale	Automatically determine PU risk scores based on the documented patient data.
Choi et al [21] (2013)	• Knowledge-based Representation of knowledge<br>• Braden Scale	Automatically determine PU risk scores with the Braden scale.
Fossum et al [22,23,24] (2011-2013)	• Knowledge-based Representation of knowledge<br>• RAPS[f] and MNA[g]	Present evidence-based interventions to support care planning based on the results of measurements of both scales: RAPS and MNA.
Horn et al [25] (2010)	• Knowledge-based Representation of knowledge<br>• AHRQ, AMDA[h] guidelines and NPULS[i]	Generates weekly reports, based on CNA[j] documentation, alerting about the risk of developing PU, for intervention and the elaboration of a care plan by the multidisciplinary team.
Sharkey et al [26] (2013)	• Knowledge-based Representation of knowledge<br>• AHRQ, AMDA guidelines and NPULS	Generate reports based on the information described by the CNA, to support the decision-making of nurses and multidisciplinary team, in the daily practice of prevention of PU, also improving the documentation and knowledge of the CNA.
Olsho et al [27] (2014)	• Knowledge-based Representation of knowledge<br>• AHRQ, AMDA guidelines and NPULS	Generates weekly clinical decision support reports (such as nutritional status, incontinence, and recent pressure ulcer history) from CNA and nursing documentation.
Cho et al [28] (2013)	• Knowledge-based Bayesian network (BN)<br>• Multi-Purpose	Predict HAPU[l] in PU prevalence, driven by the BN model from EHR[m] data, as in the Braden Scale, guides the user in developing a patient-specific prevention plan.

Beeckman et al [29] (2013)	• Knowledge-based Representation of knowledge<br>• Review of the literature and validation by PU experts + professionals from the hospital	Generates a resident-specific protocol based on the characteristics entered the system. The protocol included recommendations with a focus on skin observation, the use of support surfaces, repositioning (frequency and postures), and heel elevation.
Khong et al [30] (2015)	• Knowledge-based Decision Tree<br>• Multi-Purpose	Generates specific recommendations for the treatment of wounds for each patient, according to the characteristics and assessments of the wound inserted by nurses in the system to support them in their daily practice.
Khong et al [31] (2017)	• Knowledge-based Decision Tree<br>• Multi-Purpose	Generates patient-specific treatments of PU to assist nurses in making evidence-based decisions based on data input in pre-defined structured clinical documentation templates.

[a]Clinical Decision Support Systems
[b]Agency for Healthcare Research & Quality
[c]Pressure ulcer
[d]Italian Nurse Association for the Study of Skin Lesions
[e]Pressure Sore Status Tool
[f]Risk Assessment Pressure Scale
[g]Mini Nutritional Assessment
[h]American Medical Directors Association
[i]National Pressure Ulcer Long-term Care Study
[j]Certified Nursing Assistant
[l]Hospital-acquired pressure ulcer
[m]Electronic Health Record
